# Deep Phylogenetic Analysis of Haplogroup G1 Provides Estimates of SNP and STR Mutation Rates on the Human Y-Chromosome and Reveals Migrations of Iranic Speakers

**DOI:** 10.1371/journal.pone.0122968

**Published:** 2015-04-07

**Authors:** Oleg Balanovsky, Maxat Zhabagin, Anastasiya Agdzhoyan, Marina Chukhryaeva, Valery Zaporozhchenko, Olga Utevska, Gareth Highnam, Zhaxylyk Sabitov, Elliott Greenspan, Khadizhat Dibirova, Roza Skhalyakho, Marina Kuznetsova, Sergey Koshel, Yuldash Yusupov, Pagbajabyn Nymadawa, Zhaxybay Zhumadilov, Elvira Pocheshkhova, Marc Haber, Pierre A. Zalloua, Levon Yepiskoposyan, Anna Dybo, Chris Tyler-Smith, Elena Balanovska

**Affiliations:** 1 Vavilov Institute of General Genetics, Russian Academy of Sciences, Moscow, Russia; 2 Research Centre for Medical Genetics, Russian Academy of Sciences, Moscow, Russia; 3 Center for Life Sciences, Nazarbayev University, Astana, Republic of Kazakhstan; 4 Department of Genetics and Citology, V. N. Karazin National University, Kharkiv, Ukraine; 5 Gene by Gene, Ltd., Houston, Texas, United States of America; 6 Gumilov Eurasian National University, Astana, Republic of Kazakhstan; 7 Faculty of Geography, Lomonosov Moscow State University, Moscow, Russia; 8 Institute of Humanitarian Research of the Republic of Bashkortostan, Ufa, Russia; 9 Mongolian Academy of Medical Sciences, Ulaanbaatar, Mongolia; 10 Krasnodar State Medical University, Krasnodar, Russia; 11 The Wellcome Trust Sanger Institute, Wellcome Trust Genome Campus, Hinxton, United Kingdom; 12 The Lebanese American University, Chouran, Beirut, Lebanon; 13 Institute Molecular Biology, National Academy of Sciences of the Republic of Armenia, Yerevan, Armenia; 14 Institute of Linguistics, Russian Academy of Sciences, Moscow, Russia; University of Florence, ITALY

## Abstract

Y-chromosomal haplogroup G1 is a minor component of the overall gene pool of South-West and Central Asia but reaches up to 80% frequency in some populations scattered within this area. We have genotyped the G1-defining marker M285 in 27 Eurasian populations (n= 5,346), analyzed 367 M285-positive samples using 17 Y-STRs, and sequenced ~11 Mb of the Y-chromosome in 20 of these samples to an average coverage of 67X. This allowed detailed phylogenetic reconstruction. We identified five branches, all with high geographical specificity: G1-L1323 in Kazakhs, the closely related G1-GG1 in Mongols, G1-GG265 in Armenians and its distant brother clade G1-GG162 in Bashkirs, and G1-GG362 in West Indians. The haplotype diversity, which decreased from West Iran to Central Asia, allows us to hypothesize that this rare haplogroup could have been carried by the expansion of Iranic speakers northwards to the Eurasian steppe and via founder effects became a predominant genetic component of some populations, including the Argyn tribe of the Kazakhs. The remarkable agreement between genetic and genealogical trees of Argyns allowed us to calibrate the molecular clock using a historical date (1405 AD) of the most recent common genealogical ancestor. The mutation rate for Y-chromosomal sequence data obtained was 0.78×10^-9^ per bp per year, falling within the range of published rates. The mutation rate for Y-chromosomal STRs was 0.0022 per locus per generation, very close to the so-called genealogical rate. The “clan-based” approach to estimating the mutation rate provides a third, middle way between direct farther-to-son comparisons and using archeologically known migrations, whose dates are subject to revision and of uncertain relationship to genetic events.

## Introduction

**Fig 1:**
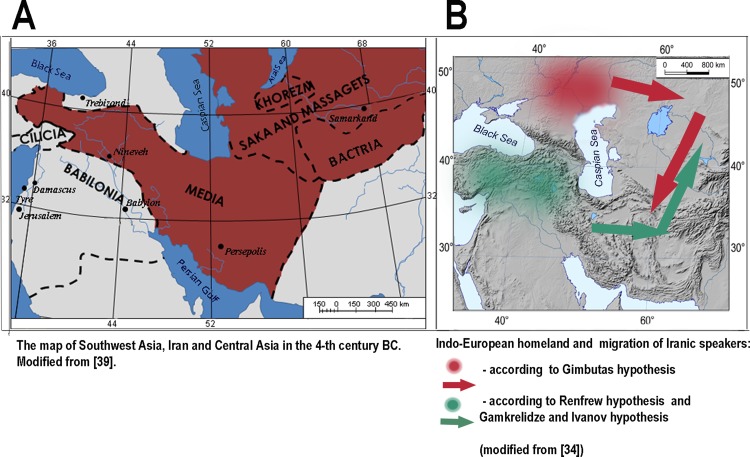
Ancient migrations of Iranic-speaking populations. A) Area populated by Iranic speakers in the middle of the first millennium BC. States whose languages belonged to the Iranic and Armenian linguistic groups are shown in red (modified from [39]). B) Homeland and migration of Iranic speakers according to the major competing theories (modified from [34]).

Despite multiple studies of the phylogeography of individual Y-chromosomal haplogroups, haplogroup G1-M285 has not received attention so far. This is partly explained by its relatively low frequency in its main area of distribution in South-West Asia [10,42], and partly by its uneven geographic distribution with a maximum frequency in the Madjar population in Kazakhstan [5]. For this reason, study of the phylogeography of haplogroup G [44] dealt mainly with the G2 sub-branch, and the only statement about G1 is an estimate of its age from Y-STR markers (19,000 ± 6,000 years). However, newly accumulated data indicate that G1 is present over a wider area in the Eurasian steppe than in Madjars only [10], and it also reaches very high frequencies in geographically distant populations of the Armenian plateau (Table 1). Thus, haplogroup G1 might mark an ancient genetic link between Iranic speakers of South-West Asia and populations of the Central Asian steppes where Iranian speech predominated in the second and first millennia BC (Fig 1A). However, the place of origin of this haplogroup remains unclear, and it is unknown whether South-West Asians and Madjars have the same or different subbranches of haplogroup G1, what the age of the branch(es) are, and which ancient migrations contributed to the contemporary distribution and diversity of this haplogroup.

**Table 1:** Frequencies of the haplogroup G1-M285 in Eurasian populations.

**Population**	**Sample size**	**G1-M285, N samples**	**G1-M285, frequency**	**latitude**		**longitude**		**country**	**locality**	**Reference**
**South-West Asia**	**5106**									
Adyghe	154	1	0.006	44,92	N	39,25	E	Russian Federation	Adygea	[52]
Armenians from Ararat Valley	110	2	0.020	40,15	N	44,18	E	Armenia	Ararat Valley	[26]
Armenians from Erzurum	99	3	0.030	39,54	N	41,16	E	Turkey	Erzurum	this study
Armenians from Gardman	96	1	0.010	40,41	N	46,21	E	Azerbaijan	Gardman	[26]
Armenians from Iran	34	1	0.030	35,42	N	51,25	E	Iran	Tehran	[21]
Armenians (diaspora sampled in Krasnodar region)	155	19	0.123	40,99	N	39,71	E	Turkey	Trabzon	this study
Armenians Hamshenis	90	38	0.422	41,01	N	39,72	E	Turkey	Trabzon	this study
Chechens	283	1	0.003	43,25	N	45,82	E	Russian Federation	Chechnya	[2,52]
Azeri	21	1	0.050	38,68	N	47,38	E	Iran		[10]
Georgians	64	1	0.016	42,14	N	43,57	E	Georgia		this study
Iranians (Gilan)	91	3	0.033	36,96	N	49,62	E	Iran	Gilan	[10, 21]
Iranians (Kordestan)	25	1	0.040	35,09	N	47,23	E	Iran	Kordestan	[10]
Iranians (south-east)	358	18	0.051	29,72	N	56,11	E	Iran		[24,33,42]
Kabardinians	371	2	0.005	43,41	N	43,32	E	Russian Federation	Kabardino-Balkaria	this study; [52]
Saudi Arabians	157	1	0.006	24,70	N	46,70	E	Saudi Arabia		[1]
Turks (North-Eastern)	80	5	0.063	40,80	N	38,60	E	Turkey		[9]
United Arab Emirates	163	4	0.025	24,28	N	54,22	E	United Arab Emirates		[7]
Jordanians	286	3	0.011	30,92	N	36,29	E	Jordan		this study
Lebanese	1425	12	0.008	33,84	N	35,81	E	Lebanon		this study
Syrians	566	3	0.005	35,09	N	38,47	E	Syria		this study
Assyrian	39	2	0.051	37,90	N	45,69	E	Iran	Azarbaijan Gharbi	[21]
Persian	44	1	0.023	29,37	N	52,32	E	Iran	Fars	[21]
Bandari	131	4	0.031	27,18	N	56,27	E	Iran	Hormozgan	[21]
Persian	59	1	0.017	36,29	N	59,60	E	Iran	Khorosan	[21]
Kurd	59	2	0.034	35,64	N	46,87	E	Iran	Kurdestan	[21]
Lur	50	1	0.020	33,48	N	48,35	E	Iran	Lurestan	[21]
Mazandarani	72	3	0.042	36,56	N	53,05	E	Iran	Mazandaran	[21]
Baluch	24	1	0.042	28,53	N	64,25	E	Iran	Balouchestan	[21]
**Central Asia**	**1841**									
China (Inner Mongolia and Ningxia)	151	2	0.016	37,53	N	105,91	E	China	Ningxia; Inner Mongolia	[27,51,55]
Kazakhs (Kerbulaksky)	134	2	0.015	44,33	N	78,43	E	Kazakhstan	Kerbulak, Almaty	this study
Kazakhs (Katonkaragaysky)	130	2	0.015	49,17	N	85,60	E	Kazakhstan	Katonkaragay, East Kazakhstan	this study
Kazakhs (Zharminsky)	101	3	0.030	49,80	N	81,27	E	Kazakhstan	Zharma, East Kazakhstan	this study
Kazakhs (Moiynkumsky)	108	6	0.056	44,42	N	71,59	E	Kazakhstan	Moiynkum, Jambyl	this study
Kazakhs (Karkaralinsky)	178	94	0.528	49,40	N	75,47	E	Kazakhstan	Karkaraly, Karagandy	this study
Kazakhs (Amangeldinsky)	141	36	0.255	52,35	N	65,04	E	Kazakhstan	Amangeldi, Kostanay	this study
Kazakhs (Akzharsky)	90	50	0.556	53,31	N	71,36	E	Kazakhstan	Akzhar, North Kazakhstan	this study
Kazakhs (Magzhan Zhumabaev)	87	30	0.345	54,45	N	70,26	E	Kazakhstan	Magzhan Zhumabaev, North Kazakhstan	this study
Kazakhs (Arysky)	118	8	0.068	42,43	N	68,80	E	Kazakhstan	Arysky, South Kazakhstan	this study
Kazakhs Madjar	45	39	0.867	49,56	N	64,00	E	Kazakhstan	Taush, Torgay area	[5]
Kirghiz (Pamirs)	106	1	0.009	38,15	N	73,95	E	Tajikistan	Gorno-Badakhshan Autonomous Province	this study
Mongols Khalkh (Setsen khan)	68	1	0.015	48,00	N	113,00	E	Mongolia	historical aimak Setsen	this study
Mongols Dariganga	73	4	0.055	47,13	N	114,47	E	Mongolia	Dornod and Sükhbaatar Provinces	this study
Mongols Uuld	41	1	0.024	48,95	N	91,16	E	Mongolia	Bayan-Ölgii Province	this study
Mongol-SouthEast	23	1	0.040	45,87	N	113,04	E	Mongolia		[10]
Tajiks from Afghanistan	56	1	0.020	35,94	N	69,96	E	Afghanistan		[23]
Tajiks Mountain	85	1	0.012	39,37	N	68,52	E	Tajikistan	Aininsky district	this study
Tajiks-Badakhshan from Afghanistan	37	1	0.030	37,11	N	70,84	E	Afghanistan		[10]
Tajiks-Takhar from Afghanistan	35	1	0.030	36,70	N	69,45	E	Afghanistan		[10]
Pashtun-Baghlan	34	1	0.030	36,29	N	68,29	E	Afghanistan		[10]
**South Asia**	**402**									
Brahui	25	1	0.040	29,02	N	62,84	E	Pakistan		[10]
Gujarat	185	2	0.011	22,78	N	71,90	E	India	Gujarat	[12,31], 1000 Genomes project
Lingayat	101	1	0.010	12,97	N	77,56	E	India	Karnataka	[8]
Pakistan (south)	91	1	0.011	26,35	N	68,00	E	Pakistan		[47]
**Europe**	**1293**									
Bashkirs (Ancient tribes)	87	1	0.011	52,59	N	58,06	E	Russian Federation	Bashkortostan Republic	this study
Bashkirs (Kipchak tribes)	125	15	0.120	52,40	N	56,33	E	Russian Federation	Bashkortostan Republic	this study
Crimean Tatars	323	2	0.006	45,00	N	34,00	E		Crimea	this study
Italians	193	4	0.020	42,05	N	13,42	E	Italy	different regions	[6]
Russians (Ryazan)	195	2	0.010	53,93	N	40,68	E	Russian Federation	Ryazan region	this study
Russians (Vologda)	121	2	0.017	59,38	N	39,15	E	Russian Federation	Vologda region	[3]
Ukrainians (Rovno)	100	1	0.010	51,32	N	26,58	E	Ukraine	Rovno region	this study

These details of haplogroup G1 phylogeography have been hard to answer, because existing methods allowed only slow progress in discovering phylogenetically informative SNPs. Fortunately, during recent years the possibility for full resequencing of the Y-chromosome [17,41,43,49,50], and more particularly the Y-capture technologies which became commercially available in the year 2013, stimulated intensive discovery of phylogenetically informative SNPs. For example, during the last decade (from the first extensive papers in 2000 till 2011) only 485 SNPs were placed on the global Y-chromosomal phylogenetic tree, while in the three following years the number of SNPs has exceed 9,000 (www.isogg.org).

Within the last decade, there has been significant uncertainty in dating Y-chromosomal haplogroups due to a three-fold difference between so-called “genealogical” and “evolutionary” mutation rates of Y-STRs. The former rates were repeatedly obtained in a set of studies [18,22,46] comparing father-son pairs, while the latter was obtained in single study [54] where calibration was done using population events with known historical dates. Increasing datasets of complete Y-chromosomal sequences allowed new calculations of the mutation rates, this time focused on SNPs. Four mutation rates have been suggested so far, ranging from 0.6 to 1.0 ×10^-9^ per bp per year: the pedigree-based rate [50], calibrations based on peopling of the Americas [41] and Sardinia [17], and the rate adopted from the pedigree rate for autosomal SNPs [37]. The two-fold difference between these rates makes further estimations necessary. In the current study we had the chance to calibrate the Y-chromosomal molecular clock using a historically reliable date of the most recent common genealogical ancestor of carriers of haplogroup G1 in Kazakh clans.

Migration of Iranic-speaking populations between the Central Asian steppes and South-West Asian uplands is an important issue in human population history, directly related to the much-debated problem of the homeland and early migrations of Indo-Europeans. Followers of the Kurgan theory propose that the carriers of Iranic languages expanded from the Eurasian steppe southward to present-day Iran, from which region these languages received their name (Fig 1B). The competing theory locating the Indo-European homeland in Eastern Anatolia proposes that the Iranic branch migrated from the Iranian plateau northward to the steppes (Fig 1B). Thus, both theories agree on the area populated by ancient Iranic-speakers (both the Iranian-Armenian plateau and Central Asia steppes) and later replacement of Iranic languages in the steppes by the Turkic ones. But these theories suggested opposite directions of the population movements between the steppes and uplands [34].

This study presents a deep phylogeographic analysis of haplogroup G1 by combining traditional approaches with the new powerful options emerging from complete sequencing of the Y-chromosome. We set out to provide a new independent estimate of the mutation rate using the tight links between haplogroups and clans typical in patrilineal nomadic societies. In addition, we aimed to find which direction of the ancient migration of Iranic speakers better fits the haplogroup G1 phylogenetic pattern.

## Methods

### Genotyping

We genotyped the commonly-used SNP M285 which defines haplogroup G1 (YCC, 2002) in multiple Eurasian populations using the TaqMan technique (Applied Biosystems) and identified 367 M285-derived samples in 27 populations. All these samples were then genotyped at 17 Y-chromosomal STRs using the Y-filer genotyping kit (Applied Biosystems). All sample donors gave their written informed consent (the study was approved by the Ethics Committee of the Research Centre for Medical Genetics, Russian Academy of Medical Sciences). Data available from the literature were also incorporated (Table 1, S1 Table).

Then we selected 19 samples for high-throughput sequencing of the Y-chromosome. To capture maximum phylogenetic diversity and thus increase the cost-effectiveness of the analyses, we applied three criteria for selecting samples. The geographic criterion led to samples from both steppe and mountain parts of the haplogroup’s area being included, particularly from populations where G1 frequency is high. The phylogenetic criterion led to samples from all clusters revealed on the STR network being included and represented by at least two samples for full sequencing, because STR-clusters might reflect real phylogenetic branches and a single sample would not allow us to distinguish phylogenetically-informative SNPs from private ones. The third criterion could be applied only to those populations where paternal clan structure is present: it led to representatives from different clans being included because members of the same clan have a high probability of sharing almost identical paternal lineages. As an outgroup for the 19 G1 samples, we also sequenced one sample from its brother haplogroup, G2.

Y-chromosomal genotyping was performed using a custom enrichment design created for the commercially available “BigY” product offered by Gene By Gene, Ltd. In total, the target regions attempt to sequence around 20 million base pairs with 67,000 capture probes, on the Illumina HiSeq platform. This design captured 11,383,697 bp within the non-recombining male-specific Y-chromosome, consistent with regions genotyped by previous Y sequencing studies [41] and the Y positions placed on the phylogenetic tree by the Y Chromosome Consortium [28]. Following BigY sequencing, and also as part of the product, downstream software analysis was performed using the Arpeggi Engine (AEngine) pipeline. This includes short read mapping, alignment post-processing, and variant calling. For quality control purposes, BigY samples are monitored for read totals, average coverage and average base quality, and should a sample fall below BigY standard thresholds, the sample is re-run. A regions file listing the genomic build 37 capture targets of BigY can be found at https://www.familytreedna.com/documents/bigy_targets.txt. Variants found across the samples are classified as any deviation from the reference genome, and in addition, we reported genotypes for about 37,000 phylogenetically informative SNPs in the FamilyTreeDNA database (www.familytreedna.com).

In addition to the genotyping per sample, we wanted to ensure for this study that SNP positions examined were adequately covered across all samples. This is a concern, because many variant calling methods in high-throughput sequencing are ambiguous when not reporting a variant as to whether there was not enough coverage to genotype, or if there was a legitimate homozygous reference genotype. To discern such cases, each BigY sample was given a “confidence” region list determined by genotype quality scores for each base. The genotype quality is computed as the probability that the genotype is correct, according to a phred score. This probability is derived from AEngine’s proprietary statistical model considering characteristics of read coverage, individual read mapping qualities, and base sequencing quality scored by the HiSeq. A base position is appended to the confidence regions for that sample if its genotype quality score is above 3.02. Thus, if there is no variant occurring at a base within confidence intervals for a sample, it can be assumed that the sample is reference genotype at that position. Variant calls were produced and handled as Variant Call Format (VCF) files, according to the established field standards (http://samtools.github.io/hts-specs/VCFv4.1.pdf). To this effect, the intersection of confident regions covered by the 20 samples studied was also recorded, and can be found within the Supplementary Data. More details on the BigY capture sequencing method are available at https://www.familytreedna.com/learn/wp-content/uploads/2014/08/BIG_Y_WhitePager.pdf, which features the methods and the capture probe list and target regions.

To estimate the potential sequencing error rate, we applied a phylogenetic approach. We checked whether we found all SNPs in the BigY captured region which are known to be phylogenetically located between haplogroups A0 and G (www.isogg.org) and thus should be present in our samples. The proportion of missed SNPs was the false negative rate. We also checked whether we see SNPs known to define other haplogroups, which are therefore not expected to be present in our haplogroup G samples. The proportion of these unexpected SNPs was considered as the false positive rate. We note that this approach overestimates the error rate, because it considers parallel mutations as errors and ignores potential inaccuracies in identifying the SNP ancestral states in ISOGG database. The (over)estimated rates were 0.008 for false negatives and 0.005 for false positives. (See details in S2 Table).

### Analyses based on G1 frequencies and STR-haplotypes

**Fig 2:**
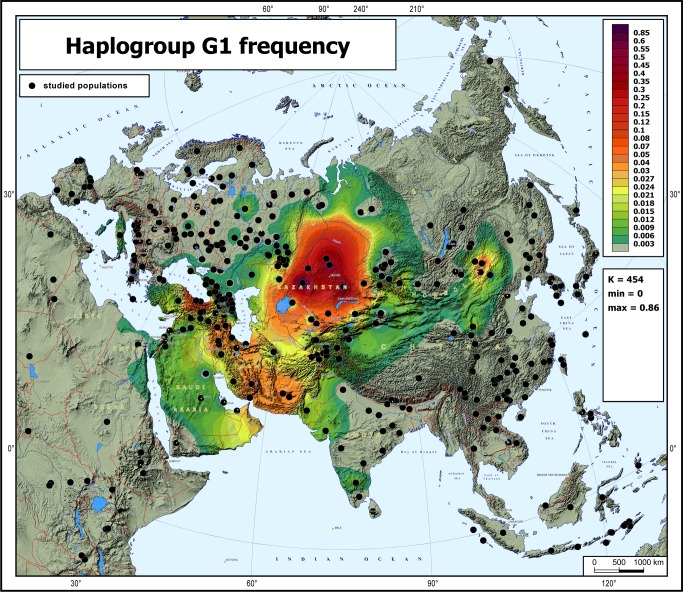
Frequency distribution map of haplogroup G1. The black points represent the populations analyzed. Abbreviations in the statistical legend indicate the following: K, number of the populations studied; MIN and MAX, the minimal and maximum frequencies on the map.

The frequency distribution map of haplogroup G1-M285 was created using data reported here for the first time (27 populations, Table 1), data from the literature (33 populations, Table 1) and published data on other 266 Eurasian populations where G1 frequency was zero. The map was created in the GeneGeo software as described previously [2,32] setting the weight function to 2 and radius of influence to 2500 km. The map is presented at two scales: a specific scale highlighting the distribution pattern of this haplogroup (Fig 2) and the “universal” scale routinely used in GeneGeo for mapping haplogroup frequencies (S1 Fig).

An analysis of molecular variance (AMOVA) was performed using Arlequin [13] on two groups of populations: those from the ancient area of Iranic speakers, compared to the group of all other Eurasian populations. We calculated variation among these two groups of populations using data on each haplogroup separately and identified haplogroups demonstrating highest differentiation between “Iranic” and “non-Iranic” populations (S3 Table).

Reduced median networks [4] of STR haplotypes (S1 Table) were created in the programs Network 4.6.0.0 and Network Publisher (Fluxus-Engineering, http://www.fluxus-engineering.com). We applied the preprocessing star contraction algorithm [16] and postprocessing Steiner maximum parsimony algorithm [40]. The allele sizes for locus DYS389II were determined with the subtraction of DYS389I. Loci DYS385a and DYS385b were excluded from network analyses. The main network was based on 15 STRs genotyped in 386 samples (S1 Table). To include data from the Madjar subclan of the Kazakhs [5], the second network restricted to the Argyn Kazakh population was based on 10 STRs (S1 Table).

Haplotype diversity was calculated according to [38] as $HD=1-\sum p_{i}^{2}$, where *p*_*i*_ is the frequency of the *i* ^*th*^ haplotype. Data on 17 Y-STR haplotypes (DYS389II alleles were subtracted) belonging to G1-haplogroup from S1 Table were used. Neighbouring populations with small numbers of G1 haplotypes were pooled to reach a minimum sample size of six; the average sample size was 30. The values of haplotype diversity were plotted on a map using the GeneGeo software with the weight function set to 4.

### Phylogenetic analysis of the Y-chromosomal sequence data

The BigY output VCF files (S1 Data) contained around 33,700–35,900 SNP calls in each of 20 samples. We combined these datasets into single table and filtered out (i) indels, (ii) SNPs with a call rate below 95% (i.e. not called in at least one out of 20 samples; BED files indicating called ranges for each sample are present in the S1 Data) and (iii) SNPs which demonstrated no polymorphism in our samples (i.e. all samples were either identical to or all were different from the reference at these positions). The resulted *filtered dataset* (S4 Table) consisted of 19 G1 samples, one G2 outgroup sample and 636 SNP positions with very little homoplasy.

The parsimony trees were constructed from this dataset using TNT [20] and Phylomurka (http://phylomurka.sourceforge.net) software. Only one optimal topology was obtained although the states of internal nodes could be marked in different ways. S5 Table presents the ages of branches estimated according to [45].

The same dataset was also subjected to analysis with BEAST software [11] which can reconstruct phylogeny and estimate divergence time by a number of Markov chain Monte Carlo methods. For the test we chose the GTR nucleotide substitution model and Gamma-distributed site heterogeneity with default parameters. We tested both strict and lognormal relaxed clock models and finally preferred the latter due to a positive posterior value of the rate variance between tree branches (S5 Table). For the tree prior we chose the Expansion Growth model assuming that the population grew exponentially since a relatively recent time. The prior for the mutation rate was set as a uniformly distributed value, initially equal to 1.2×10^-5^ per SNP (S5 Table) while the age of Kazakh cluster was forced to be normally distributed with the mean of 627 years (the value obtained using genealogical records, see below) and standard error of 50 years. Sufficient ESS values were achieved with the MCMC chain size of 20,000,000 and higher. The consensus of 10,000 trees produced by BEAST is the same as our parsimony tree, and Bayesian age estimates show less than 20% difference from those obtained with Rho statistics (S5 Table). Bayesian methods such as BEAST assume random sampling from a population and interpretation of their output can be less straightforward when lineage-based sampling is used [25], and our dataset was restricted to a single haplogroup. However, the coinciding topologies of the trees generated by the different methods in our study shows that the phylogenetic structure is robust to this concern. Note that in our analyses, choosing the model of population growth had the major influence on the results—probably larger than the influence of sampling from a particular lineage—but we report the general consensus of the results across all settings used.

In an additional analysis we included two G1 samples from the 1000 Genomes Project (NA20858 and NA20870, Gujarati Indians sampled in Houston, Texas (GIH), 2-4X average coverage). Data were handled in the same way, although the lower coverage of the 1000 Genomes samples halved the number of SNP calls and the filtered dataset consisted of 22 samples and 393 SNPs (S4 Table). The parsimony method yielded two optimal topologies, and the one supporting the monophyly of all non-Indian lineages was preferred as a more likely reconstruction.

## Results

We genotyped the haplogroup G1-specific marker M285 [28] in 5,346 individuals from 27 Eurasian populations (Table 1) and identified 367 M285-positive samples, which were further genotyped by 17 Y-STRs (S1 Table). For 19 haplogroup G1 samples and one outgroup G2 sample we performed complete sequencing of the “extended gold standard” regions of the Y-chromosome.

### The frequency distribution of the haplogroup G1

The frequency distribution of haplogroup G1 in Eurasia is presented in Fig 2, which is based on the dataset from Table 1. This haplogroup is distributed over a large area from Italy in the west to Mongolia in the east, but is present at high frequencies only in an uninterrupted area including the Central Asian steppes and Iranian-Armenian plateau. Two frequency peaks can be seen at the opposite sides of this area, namely in North Kazakhstan (up to 80%) within the steppe part and in Armenia (up to 42%) within its mountainous part. In Kazakhs, haplogroup G1 is typical of the Argyn tribe: among 291 G1 samples with known tribal affiliation in Kazakhs, 262 (90%) belong to the Argyn tribe. In Armenians, this haplogroup is particularly frequent in Hemsheni Armenians (42%). Both populations are not small: according to a census performed in the beginning of the 20^th^ century—tribal affiliation was not recorded in later censuses—there were around 500,000 Argyns [36] and now the population is expected to be larger; the estimated present-day number of Hemsheni Armenians is 150,000 [35]. Thus, the increased frequency of G1 cannot be explained by recent genetic drift and likely indicates drift during the formation of these populations many centuries ago.

It is notable that the area of haplogroup G1, including the Eurasian steppes from the North Black Sea region to the Mongolian Altai and South-Western Asian uplands (Iran and historical Great Armenia), corresponds well with the area populated by Iranic speakers in the second and first millennia BC (Fig 1A). This correspondence was statistically confirmed by AMOVA (S3 Table).

### STR-variation within haplogroup G1

**Fig 3:**
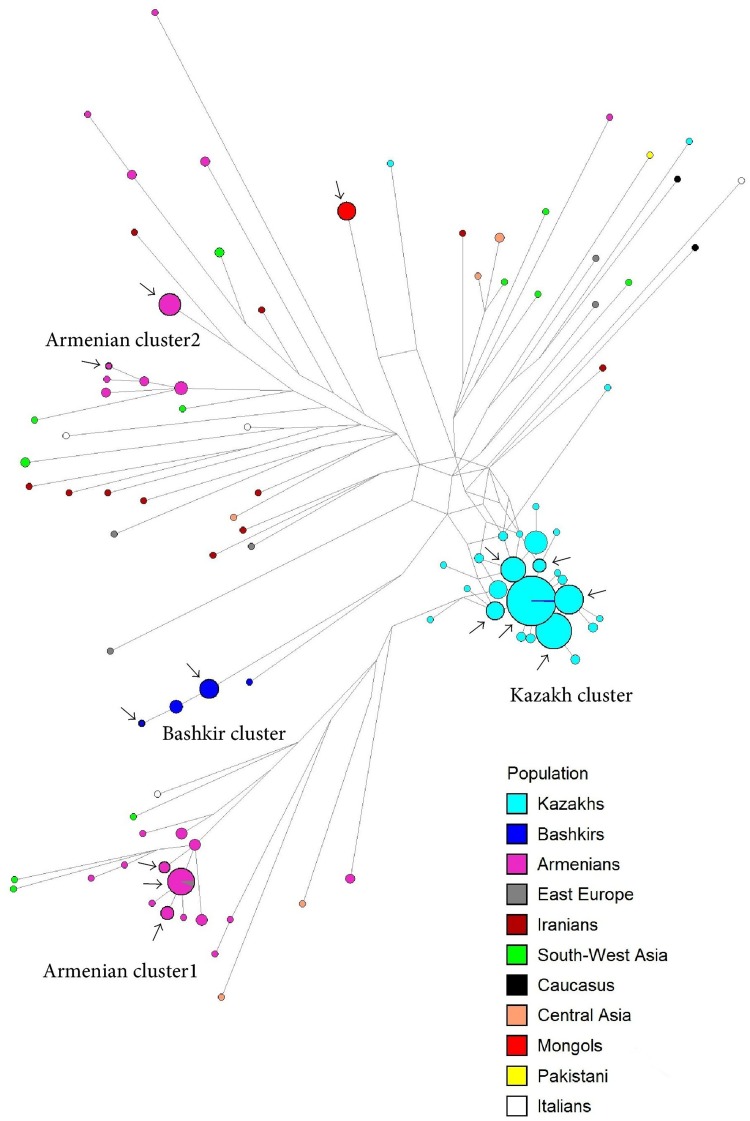
Network of Y-STR haplotypes within haplogroup G1. Arrows mark samples chosen for Y-chromosomal sequencing.

On the network (Fig 3) four clusters are visible: two include only Armenian samples, while other two are specific to Kazakhs and Bashkirs, respectively. Samples from other populations are spread all around the network and do not form clusters. The Kazakh cluster is highly specific to the Argyn tribe within the Kazakhs; the Armenian cluster includes more Hemsheni Armenians than other Armenian populations; all but one members of the Bashkir cluster belong to the Kangly tribe of Bashkirs. The Y-STR pattern thus shows that haplogroup G1 is genetically diverse and widespread, with some sub-branches at high frequencies due to founder effects, while others remain at very low frequencies in occasional locations within the area of the haplogroup.

**Fig 4:**
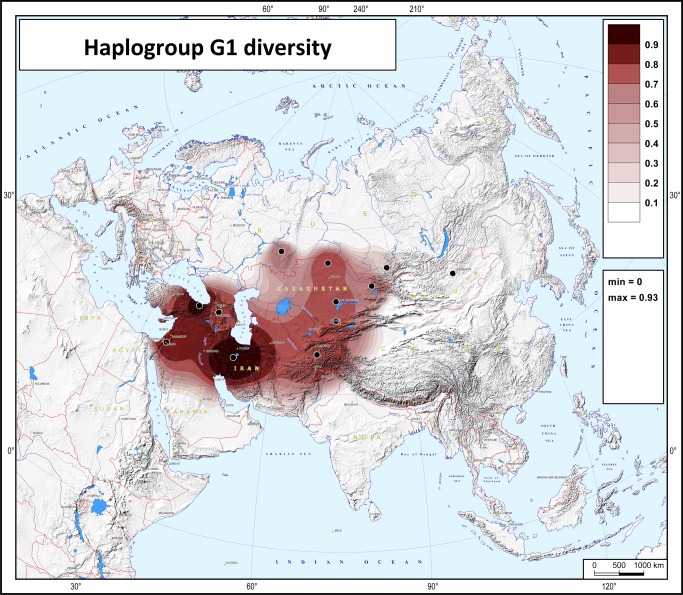
Map of haplotype diversity of haplogroup G1. The black points represent the populations for which diversity values were calculated. Abbreviations in the statistical legend indicate the following: MIN and MAX, the minimal and maximum values on the map.

The haplotype diversity of haplogroup G1 varies drastically from 92% in Iran to zero in Mongolia (Table 2). The map (Fig 4) reveals a cline of decreasing diversity from West Iran to the eastern parts of South-West Asia and further northward to the Eurasian steppes.

**Table 2:** Haplotype diversity of haplogroup G1-M285 in South-Western and Central Asian populations.

**Population**	**N**	**N**_HT_	**F**_MAX_	**HD**	**Reference**
Iranians and Azeris (Iran)	16	15	0.125	0.9297	this study
Armenians (Turkey)	60	31	0.250	0.9056	this study
Lebanese and Jordanians	8	7	0.250	0.8438	this study
Kazakhs (North Kazakhstan)	116	35	0.448	0.7794	this study
Tajiks (Afghanistan, Tajikistan)	6	5	0.333	0.7778	this study
Armenians (Armenia)	7	5	0.286	0.7755	this study
Kazakhs (Central Kazakhstan)	100	26	0.490	0.7394	this study
Kazakhs (South Kazakhstan)	14	8	0.500	0.7143	this study
Bashkirs (Russia)	15	6	0.467	0.6933	this study
Kazakhs (East Kazakhstan)	9	4	0.444	0.6667	this study
Kazakhs (Altaian)	6	2	0.833	0.2778	this study
Mongols (Mongolia)	7	1	1.000	0.0000	this study

N—number of G1 samples genotyped by 17 Y-STRs;

N_HT_—number of different Y-chromosomal STR haplotypes;

F_MAX_—frequency of the most frequent haplotype;

HD—haplotype diversity; the populations were sorted according to the level of HD.

### A sequence-based phylogenetic tree of haplogroup G1

We sequenced ~11 Mb of the Y-chromosome in 19 samples selected using three criteria to cover the maximum diversity within haplogroup G1. The average coverage was 67x, ranging from 48x to 88x. Among the 766 SNPs in the filtered dataset (see Methods for details) 281 have already been listed by ISOGG (www.isogg.org) and/or YFULL (www.yfull.com), most of these SNPs defined our outgroup G2 sample. We gave the remaining SNPs (S4 Table) names from GG001 to GG388 (GG is the abbreviation of Gene Geography).

The phylogenetic trees created by parsimony (S2 Fig) and Bayesian approaches (S3 Fig) coincided fully—not surprisingly, since the full-Y-chromosomal dataset allows robust reconstruction of phylogenetic events. The trees reveal three principal clusters: Kazakh, Armenian and Bashkir, with 100% specificity of the cluster members to the corresponding populations. The Armenian and Bashkir clusters have a shared ancestor on the tree, while the Kazakh cluster and an Indian cluster (described below) form independent branches. The Mongol sample forms a branch on its own, although this Mongolian branch then joins the Kazakh cluster in agreement with common geographical and historical background of the two groups.

This tree corresponds in general with the pattern revealed by the STR-based network (Fig 3). Kazakh, Armenian and Bashkir clusters are clearly visible on both plots. However, Armenians, which seemed to have two clusters and multiple single-haplotype mini-branches from the STR data, all turned out to belong to one single and compact cluster when complete Y-chromosomal resequencing was performed. Similarly, the Mongolian sample, which seemed to form a separate branch on the STR-based plot, actually joins the Kazakh cluster. We conclude that haplogroup G1 lineages actually form a restricted number of clusters, in contrast to the impression one can get from STR-data, with the caveat that the number of sequences examined thus far is limited.

**Fig 5:**
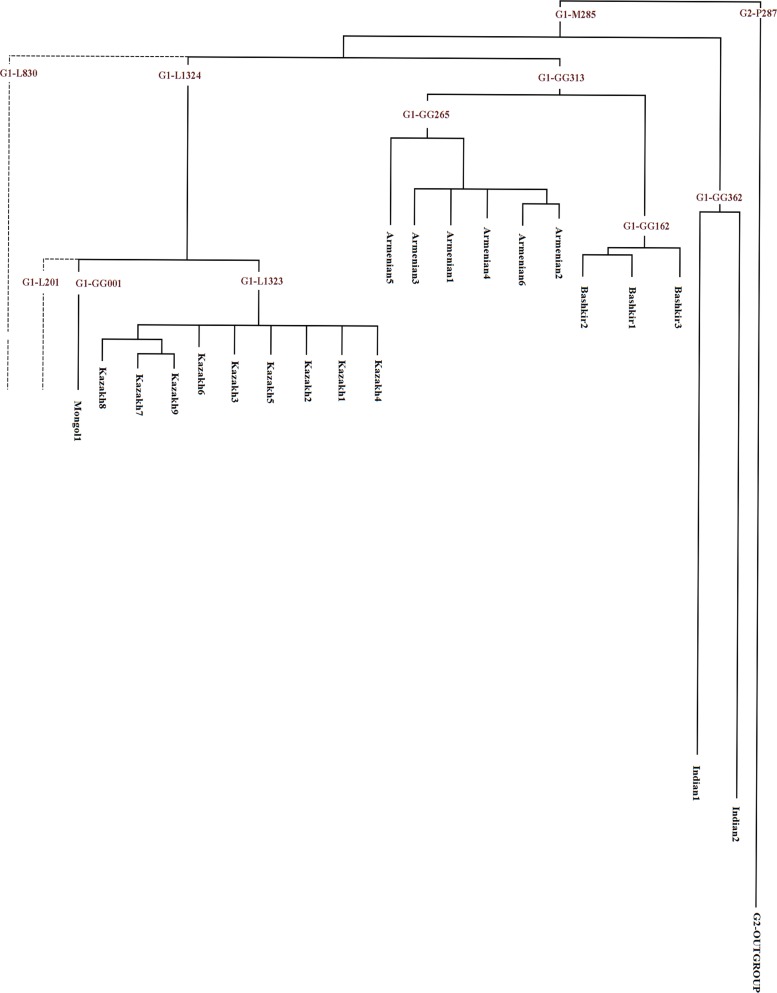
Y-chromosome haplogroup G1 phylogeny. The tree combines the high-coverage dataset reported in this study with data from 1000 Genomes Project. Dotted lines indicate the approximate phylogenetic position of two previously reported G1 branches which were absent among our samples.

The presence of additional clusters was confirmed when we included two GIH (Gujarat Indians from Houston) samples from the 1000 Genomes Project, which are the only publicly available data on haplogroup G1. Including the low coverage sequences halved the number of SNPs called in all samples (S4 Table), but tree revealed the same topology, and the Indian G1s formed their own cluster (Fig 5). One technical point is notable: the lengths of all the branches on the tree are similar, as they should be if the mutation rate is constant. The only exception is the very long branches of the samples from the 1000 Genomes Project, which is likely caused by the filtering criteria not being optimized for low coverage datasets. However, 26 SNPs were independently called in both samples, thus confirming the reality of the Indian-specific branch of haplogroup G1.

The Kazakh cluster fits the previously described G-L1323 branch (www.isogg.org), while the Bashkir, Armenian, Mongolian and Indian branches were not previously reported. Fig 5 approximates the phylogenetic relations between five branches found in our study and three previously known ones.

### Estimating the mutation rate

**Fig 6:**
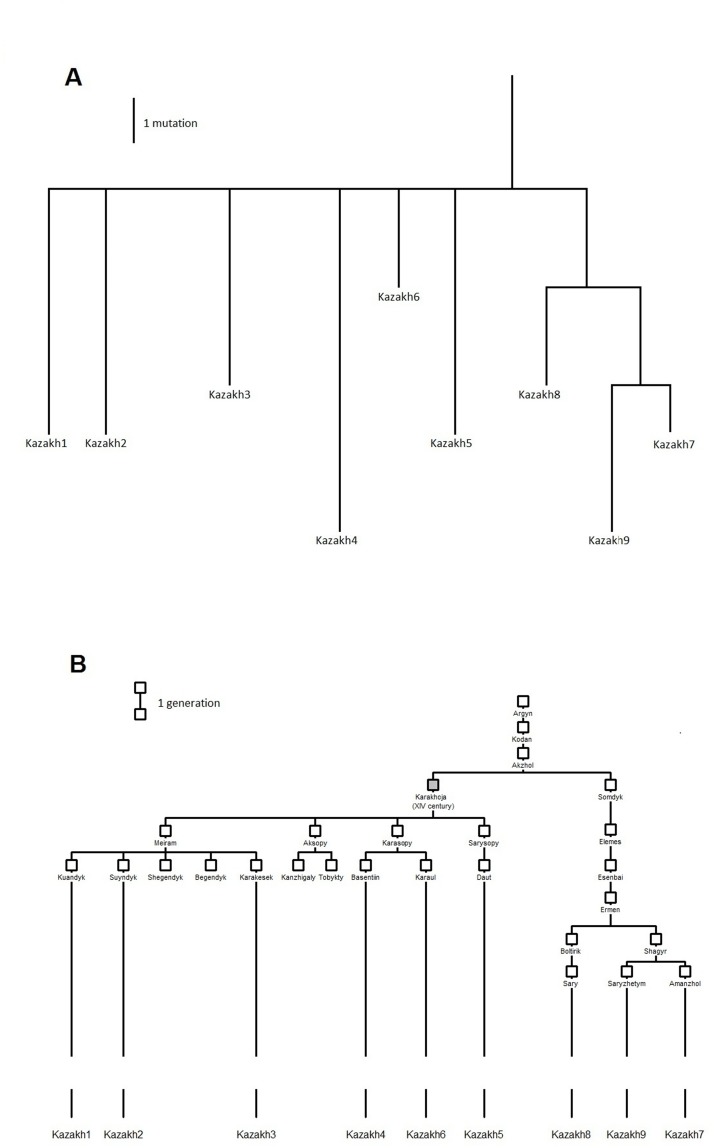
Genetic and genealogical reconstructions of the relationship between members of the Argyn tribe of the Kazakh: A) Genetic tree reconstructed from Y-chromosome sequences of the Kazakh samples. B) Genealogical tree of the Argyn tribe of the Kazakh. Each sequenced Kazakh sample is attributed to the clan it originates from. The genealogical ancestor with the known historical date is marked in grey.

The Argyn tribe in which haplogroup G1 predominates is believed to descend from a single male common ancestor (Argyn) and is divided into 12 clans (Fig 6B). Though there is no historical evidence for the existence of Argyn, who is known only from genealogical tradition, his great-grandson Karakhoja is a historical personality and is mentioned, among other sources, as ambassador of the Toshtamish khan—ruler of the Golden Horde—to Tamerlane in 1405. Most of the Argyn clans are believed to originate from Karakhoja (S4 Fig) and other clans are believed to originate from his brother Somdyk.

The genetic tree based on high-throughput sequencing of the Kazakh G1 chromosomes (Fig 6A) perfectly fits the genealogical tree: representatives of Argyn clans who originated from Karakhoja (from Kazakh1 to Kazakh6) form a single and young subcluster and all are equidistant from the MRCA, as predicted by the genealogy (Fig 6B). Thus, the *de jure* ancestor known from genealogical tradition and historical records was likely to be also the *de facto* biological ancestor of most present-day male members of the Argyn tribe. Considering the time span of 606 years between Karakhoja (who was around 50 years old in 1405 and then likely fathered his sons on average around 1385) and average date of birth of the 6 present-day Kazakhs sampled (1991), the total length of Y-chromosomal segments sequenced in each of these 6 Kazakh samples (10,005,352 bp), and the average number of accumulated mutations 4.67 (S2 Fig), we obtained the mutation rate for Y-chromosomal sequences 0.77×10^-9^ per bp per year.

While this paper was under review, we obtained experimental data from three additional samples, representing clans claiming their origin from Karakhoja’s brother Somdyk (Kazakh 7, Kazakh8, and Kazakh9, Fig 6B). These samples formed a subcluster of their own (Fig 6A), a genetic finding which fully agrees with the genealogical tradition. This allows us to reconstruct even minor details, like the observations that mutations 23081087 C->T and 23526483 A->G occurred between Akzhol and his great-great-grandson Ermen, and that no mutations occurred between Akzhol and his son Karakhoja within the sequenced regions. Considering the time span of 627 years between Akzhol (we added 30 years—the typical male generation time—to the time estimated above for Karakhoja) and the average date of birth of the 9 present-day Kazakhs sampled (1982), the total length of Y-chromosomal segments sequenced in each of these 9 Kazakh samples (9972660 bp), and the average number of accumulated mutations (4.89: S2 Fig), we obtained a SNP mutation rate for Y-chromosomal sequences of 0.78×10^-9^ per bp per year. This value agrees well with the aforementioned initial estimation.

We applied the same approach to Y-STR data also. Including data on 15-Y-STR haplotypes in Argyns (S1 Table, Fig 3), we counted 21 generations (using a male generation time of around 30 years [14]) and 0.68 mutations on average. Thus we obtained the mutation rate for Y-STRs 0.0022 per locus per generation. It is very close to the “genealogical” rate [18,22,46], despite the time span used (600 years) better fitting the population events used for estimating the “evolutionary” rate [54].

## Discussion

The pattern of geographic distribution of haplogroup G1-M285 is to some degree exceptional, as it cannot be called either a West-Eurasian or an East-Eurasian lineage (Fig 2). Instead, its spread zone corresponds well with the area of ancient Iranic-speaking groups who dwelled both on the Iranian plateau (and neighboring uplands) and Eurasian steppe. The increased dataset on G1 frequencies and STR-variation leaves little doubt that G1 is partitioned into a small number of clusters (branches), each frequent in a particular population. It became very clear from the phylogenetic tree based on full Y-chromosomal sequences that the geographic specificity of G1 branches is virtually absolute, as all five branches are specific, respectively, to West Indians, Kazakhs, Mongols, Bashkirs, and Armenians, although further sampling in Iran and Central Asian countries may reveal additional minor branches.

The question arises of whether the homeland of G1 was in steppe or mountains. Much higher STR variation in the west part of the Iranian-Armenian plateau makes the mountain homeland a more probable candidate. This conclusion fits the Anatolian theory of Indo-European origins, and the pattern of STR diversity (Fig 4) fits especially well. Migrations from Iran to Central Asia are also clear from paleoanthropological data [19,29]. Though haplogroup G1 certainly cannot serve as a marker for the Indo-European expansion in general, this haplogroup might be a genetic component carried by a wave of Iranic-speaker migration and brought northward to the Eurasian steppe. The genetic dates suggest that all principal branches already existed when this migration started. Indeed, even the last split into the Bashkir and Armenian clusters is dated back to 8000 YBP (S5 Table), while the Armenian linguistic branch separated around 4600 YBP and Indo-Iranian languages separated around 4200 YBP (http://starling.rinet.ru/new100/eurasia_long.jpg). Haplogroup G1 might remain a minor genetic component among many Eurasian and particularly Iranic-speaking populations (as it is now rare, for example, in Italy and West India, though more frequent in its possible homeland in Iran/Armenia). When Turkic languages replaced Iranic ones in the steppes (perhaps starting from the middle of the 1^st^ millennium AD) the G1-carriers were probably assimilated into expanding Turkic and then Mongolian-speaking populations. In more recent times, haplogroup G1 has undergone three independent expansions in different geographic regions, shown by the full-Y-chromosomal analysis (Fig 5).

The expansion in Kazakhs is genetically dated to an interval of 470–750 YBP, using the range of published mutation rate point estimates [17,37,41,50]. The genealogical ancestor of the Argyn Kazakh (the main carriers of this haplogroup) lived 600 years ago, which lies in the middle of this range. Expansion from a single man to half a million descendants within 500 years (20 generations) is not really surprising. Indeed, having two surviving sons in every generation gives half a million descendants in the 19^th^ generation and Kazakh families had 3.5 children on average [36]. Also, an even more impressive expansion up to 16 million descendants was suggested for the same medieval steppe societies [53]. Note that the traditional genealogical partitioning of Argyns into three clans corresponds well with the Y-STR data (S4 Fig). This finding also questions the hypothesis [5] about the relationship between the Argyn subclan Madjars and Magyars (Hungarians), because haplogroup G1 (comprising 82% of the Madjar gene pool) finds its place within other Argyn Kazakh (S4 Fig) while no G1 samples have been reported in Hungarians so far.

The expansion in the Hemsheni Armenian is genetically dated to 1150 YBP using our rate (S5 Table). It corresponds well with the historical evidence [30,48] that the Hemsheni originated from relatives and servants of Prince Shapuh Amatuni, who migrated in 791 from the Abbasid Persian state.

The expansion in the Kangly tribe of Bashkirs is genetically dated to the 15^th^ century AD (S5 Table). This tribe originated from the Pechenegs around the 8^th^ century AD, then joined the Bashkirs, and later expansion in a restricted part of the tribe might have been caused by demographic changes when it became part of the Golden Horde in the 14^th^ century and part of the Russian state in 16^th^ century.

We note that despite geographic proximity, the ancestor of the G1 cluster in Bashkirs had no close genetic relationship to the corresponding ancestor in Kazakhs. These branches (and the third branch detected in Mongolians) have survived in the Eurasian steppe perhaps since the Scythian epoch.

The remarkable coincidence between the genealogical tree of the Argyn Kazakh clan (Fig 6B) and the genetic tree obtained from full Y-chromosomal sequences (Fig 6A) allowed us to suggest an independent calibration of the mutation rate of Y-chromosomal SNPs. This “clan” rate has been tested only within the time frame in which it was obtained (a few centuries), and in cases when it is reasonable to suppose expansion of a single paternal line rather than multiple lineages in the founding population, and by applying the “gold standard” portion of the Y-chromosome (that included in the BigY technology used in our study). Provided these limitations are taken into account, this “clan-based” calibration might be at least as reliable as calibrations based on archeological evidence, because archeological dates are subject to revision and of uncertain relationship to genetic events. For example, the calibration of the Y-chromosomal mutation rate in [41] is based on “archeological evidence that humans first colonized America around 15 kya” while the study that provides the commonly-used calibration of the mitochondrial DNA control region [15] relies on a “major wave of migration [which] brought one population ancestral to Amerinds from north-eastern Siberia to America 20,000–25,000 years ago”. Fortunately, despite differences in approaches, all mutation rates suggested for the “full” sequences of the Y-chromosome fall within the interval 0.6–1.0 ×10^-9^ per bp per year, and this uncertainty may be further narrowed, as we demonstrated for the haplogroup G1.

## Supporting Information

S1 Data The archive includes 20 VCF files for 20 sequenced samples, 20 BED files showing Y-chromosomal ranges where a given sample was sequenced reliably, and the BED files listing ranges intersecting between all 20 samples (used for tree construction) and between 6 Kazakh samples (used for calibrating the mutation rate). (ZIP)

S1 Fig Frequency distribution map of haplogroup G1 in the “universal” scale. This scale is typically used in the GeneGeo software for frequency distribution maps of all haplogroups, thus allowing easy comparisons of different maps. The black points represent the populations analyzed. Abbreviations in the statistical legend indicate the following: K, number of the populations studied; MIN and MAX, the minimal and maximum frequencies on the map.
(TIFF)

S2 Fig Detailed phylogenetic tree of haplogroup G1 obtained by the parsimony approach. The tree is based on the high quality filtered dataset from this study consisting of 20 samples and 636 SNPs. The Build 37 coordinates of the SNPs are shown along branches. ISOGG marker names are shown in red. Further details of these mutations are reported in S4 Table.
(PDF)

S3 Fig Phylogenetic tree of haplogroup G1 obtained by the Bayesian approach. The tree is based on the high quality filtered dataset from this study consisting of 20 samples and 636 SNPs. The tree was created in the BEAST software. The mean age estimates are shown for all branches.
(TIFF)

S4 Fig Network of Y-STRs-haplotypes and genealogy of the Argyn tribe. Data on haplogroup G1 Y-STRs in the Argyn tribe of the Kazakh clan came from both this study and [5]. 10-STRs haplotypes were used. The genealogy of the early generations of the Argyn tribe shows partitioning into clans. Members of each clan are color-coded in both network and genealogy.
(TIFF)

S1 Table Y-chromosomal STR haplotypes identified within haplogroup G1. (XLS)

S2 Table Upper limit estimates of the sequencing errors. (XLS)

S3 Table AMOVA results: in search for haplogroups differentiating populations of ancient area of Iranic speakers from other Eurasian populations. (XLS)

S4 Table The filtered dataset on Y-chromosomal SNPs in the samples analyzed in this study. (XLS)

S5 Table Ages of the branches identified within haplogroup G1. (XLS)

## References

[1] Abu-Amero KK, Hellani A, Gonzalez AM, Larruga JM, Cabrera VM, Underhill PA. Saudi Arabian Y-Chromosome diversity and its relationship with nearby regions. BMC genetics. 2009;10:59 10.1186/1471-2156-10-59 19772609 PMC2759955

[2] Balanovsky O, Dibirova K, Dybo A, Mudrak O, Frolova S, Pocheshkhova E, et al Parallel evolution of genes and languages in the Caucasus region. Molecular biology and evolution. 2011;28(10):2905–20. 10.1093/molbev/msr126 21571925 PMC3355373

[3] Balanovsky O, Rootsi S, Pshenichnov A, Kivisild T, Churnosov M, Evseeva I, et al Two sources of the Russian patrilineal heritage in their Eurasian context. Am J Hum Genet. 2008;82(1):236–50. 10.1016/j.ajhg.2007.09.019 18179905 PMC2253976

[4] Bandelt H-J, Forster P, Sykes BC, Richards MB. Mitochondrial Portraits of Human Populations Using Median Networks. Genetics. 1995;41:743–53. 10.1093/genetics/141.2.743 PMC1206770 8647407

[5] Biro AZ, Zalan A, Volgyi A, Pamjav H. A Y-chromosomal comparison of the Madjars (Kazakhstan) and the Magyars (Hungary). American journal of physical anthropology. 2009;139(3):305–10. 10.1002/ajpa.20984 19170200

[6] Boattini A, Martinez-Cruz B, Sarno S, Harmant C, Useli A, Sanz P, et al Uniparental markers in Italy reveal a sex-biased genetic structure and different historical strata. PloS one. 2013;8(5):e65441 10.1371/journal.pone.0065441 23734255 PMC3666984

[7] Cadenas AM, Zhivotovsky LA, Cavalli-Sforza LL, Underhill PA, Herrera RJ. Y-chromosome diversity characterizes the Gulf of Oman. European journal of human genetics: EJHG. 2008;16(3):374–86. 17928816 10.1038/sj.ejhg.5201934

[8] Chennakrishnaiah S, Perez D, Gayden T, Rivera L, Regueiro M, Herrera RJ. Indigenous and foreign Y-chromosomes characterize the Lingayat and Vokkaliga populations of Southwest India. Gene. 2013;526(2):96–106. 10.1016/j.gene.2013.04.074 23664983

[9] Cinnioglu C, King R, Kivisild T, Kalfoglu E, Atasoy S, Cavalleri GL, et al Excavating Y-chromosome haplotype strata in Anatolia. Human genetics. 2004;114(2):127–48. 14586639 10.1007/s00439-003-1031-4

[10] Di Cristofaro J, Pennarun E, Mazieres S, Myres NM, Lin AA, Temori SA, et al Afghan Hindu Kush: where Eurasian sub-continent gene flows converge. PloS one. 2013;8(10):e76748 10.1371/journal.pone.0076748 24204668 PMC3799995

[11] Drummond AJ, Suchard MA, Xie D, Rambaut A. Bayesian phylogenetics with BEAUti and the BEAST 1.7. Molecular biology and evolution. 2012;29(8):1969–73. 10.1093/molbev/mss075 22367748 PMC3408070

[12] Eaaswarkhanth M, Haque I, Ravesh Z, Romero IG, Meganathan PR, Dubey B, et al Traces of sub-Saharan and Middle Eastern lineages in Indian Muslim populations. European journal of human genetics: EJHG. 2010;18(3):354–63. 10.1038/ejhg.2009.168 19809480 PMC2859343

[13] Excoffier L, Lischer HE. Arlequin suite ver 3.5: a new series of programs to perform population genetics analyses under Linux and Windows. Molecular ecology resources. 2010;10(3):564–7. 10.1111/j.1755-0998.2010.02847.x 21565059

[14] Fenner JN. Cross-cultural estimation of the human generation interval for use in genetics-based population divergence studies. American journal of physical anthropology. 2005;128(2):415–23. 15795887 10.1002/ajpa.20188

[15] Forster P, Harding R, Torroni A, Bandelt H-J. Origin and Evolution of Native American mtDNA variation: A Reappraisal. Am J Hum Genet. 1996;59:935–45. 8808611 PMC1914796

[16] Forster P, Torroni A, Renfrew C, Röhl A. Phylogenetic Star Contraction Applied to Asian and Papuan mtDNA Evolution. Molecular biology and evolution. 2001;18:1864–81. 11557793 10.1093/oxfordjournals.molbev.a003728

[17] Francalacci P, Morelli L, Angius A, Berutti R, Reinier F, Atzeni R, et al Low-Pass DNA Sequencing of 1200 Sardinians Reconstructs European Y-Chromosome Phylogeny. Science. 2013;341:565–9. 10.1126/science.1237947 23908240 PMC5500864

[18] Ge J, Budowle B, Aranda XG, Planz JV, Eisenberg AJ, Chakraborty R. Mutation rates at Y chromosome short tandem repeats in Texas populations. Forensic science international Genetics. 2009;3(3):179–84. 10.1016/j.fsigen.2009.01.007 19414166

[19] Ginzburg V. Materials for the Anthropology of the ancient population of the Ferghana Valley Works of Kirghiz archaeological and ethnographic expedition. 1 Moscow 1956 p. 85–102 (In Russian).

[20] Goloboff PA, Farris JS, Nixon KC. TNT, a free program for phylogenetic analysis. Cladistics. 2008;24:774–86.

[21] Grugni V, Battaglia V, Hooshiar Kashani B, Parolo S, Al-Zahery N, Achilli A, et al Ancient migratory events in the Middle East: new clues from the Y-chromosome variation of modern Iranians. PloS one. 2012;7(7):e41252 10.1371/journal.pone.0041252 22815981 PMC3399854

[22] Gusmao L, Sanchez-Diz P, Calafell F, Martin P, Alonso CA, Alvarez-Fernandez F, et al Mutation rates at Y chromosome specific microsatellites. Human mutation. 2005;26(6):520–8. 16220553 10.1002/humu.20254

[23] Haber M, Platt DE, Ashrafian Bonab M, Youhanna SC, Soria-Hernanz DF, Martinez-Cruz B, et al Afghanistan's ethnic groups share a Y-chromosomal heritage structured by historical events. PloS one. 2012;7(3):e34288 10.1371/journal.pone.0034288 22470552 PMC3314501

[24] Haber M, Platt DE, Badro DA, Xue Y, El-Sibai M, Bonab MA, et al Influences of history, geography, and religion on genetic structure: the Maronites in Lebanon. European journal of human genetics: EJHG. 2011;19(3):334–40. 10.1038/ejhg.2010.177 21119711 PMC3062011

[25] Heath T. Understanding the importance of taxonomic sampling for large-scale phylogenetic analyses by simulating evolutionary processes under complex models [Doctoral thesis,]. Ausyin2008.

[26] Herrera KJ, Lowery RK, Hadden L, Calderon S, Chiou C, Yepiskoposyan L, et al Neolithic patrilineal signals indicate that the Armenian plateau was repopulated by agriculturalists. European journal of human genetics: EJHG. 2012;20(3):313–20. 10.1038/ejhg.2011.192 22085901 PMC3286660

[27] Karafet T, Xu L, Du R, Wang W, Feng S, Wells RS, et al Paternal Population History of East Asia: Sources, Patterns, and Microevolutionary Processes. Am J Hum Genet. 2001;69:615–28. 11481588 10.1086/323299 PMC1235490

[28] Karafet TM, Mendez FL, Meilerman MB, Underhill PA, Zegura SL, Hammer MF. New binary polymorphisms reshape and increase resolution of the human Y chromosomal haplogroup tree. Genome Res. 2008;18(5):830–8. 10.1101/gr.7172008 18385274 PMC2336805

[29] Kazarnitskiy A. The population of the Azov-Caspian steppes in the Bronze Age (anthropological essay). Gromov A, editor. St. Petersburg: Nauka; 2012 264 (In Russian) p.

[30] Khachikyan L. Pages from the history of Hamshen Armenians. Bulletin of Yerevan State University. 1969; 2:115–44 (In Armenian)

[31] Kivisild T, Rootsi S, Metspalu M, Mastana S, Kaldma K, Parik J, et al The Genetic Heritage of the Earliest Settlers Persists Both in Indian Tribal and Caste Populations. Am J Hum Genet. 2003;72:313–32. 12536373 10.1086/346068 PMC379225

[32] Koshel SM. Geoinformation technologies in genogeography In: Lure IK, Kravtsova VI, editors. Modern Geographic cartography. Moscow: Data + 2012 p. 158–66 (In Russian).

[33] Lashgary Z, Khodadadi A, Singh Y, Houshmand SM, Mahjoubi F, Sharma P, et al Y chromosome diversity among the Iranian religious groups: a reservoir of genetic variation. Ann Hum Biol. 2011;38(3):364–71. 10.3109/03014460.2010.535562 21329477

[34] Mallory J. In search of the Indo-Europeans: language, archaeology and myth.illustrations. London: Thames & Hudson; 1989 288 p.

[35] Margaryan A, Harutyunyan A, Khachatryan Z, Khudoyan A, Yepiskoposyan L. Paternal lineage analysis supports an Armenian rather than a Central Asian genetic origin of the Hamshenis. Hum Biol. 2012;84(4):405–22. 10.3378/027.084.0404 23249315

[36] Masanov N. Nomadic Kazakhs Civilization: the basics of life migratory habits of society. Almaty: Print-S; 2011 740 (In Russian) p.

[37] Mendez FL, Krahn T, Schrack B, Krahn AM, Veeramah KR, Woerner AE, et al An African American paternal lineage adds an extremely ancient root to the human Y chromosome phylogenetic tree. Am J Hum Genet. 2013;92(3):454–9. 10.1016/j.ajhg.2013.02.002 23453668 PMC3591855

[38] Nei M. Molecular Evolutionary Genetics. New York: Columbia University Press; 1987 512 p.

[39] Oransky I. Map 1. Southwest Asia, Iran and Central Asia in the VI. BC Introduction to Iranian philology. Moscow: Publishing House of Oriental Literature; 1960 p. 62–3 (In Russian)

[40] Polzin T, Daneschmand SV. On Steiner trees and minimum spanning trees in hypergraphs. Operations Research Letters 2003;31:12–20.

[41] Poznik GD, Henn BM, Yee MC, Sliwerska E, Euskirchen GM, Lin AA, et al Sequencing Y chromosomes resolves discrepancy in time to common ancestor of males versus females. Science. 2013;341(6145):562–5. 10.1126/science.1237619 23908239 PMC4032117

[42] Regueiro M, Cadenas AM, Gayden T, Underhill PA, Herrera RJ. Iran: tricontinental nexus for Y-chromosome driven migration. Hum Hered. 2006;61(3):132–43. 16770078 10.1159/000093774

[43] Rootsi S, Behar DM, Jarve M, Lin AA, Myres NM, Passarelli B, et al Phylogenetic applications of whole Y-chromosome sequences and the Near Eastern origin of Ashkenazi Levites. Nat Commun. 2013;4:2928 10.1038/ncomms3928 24346185 PMC3905698

[44] Rootsi S, Myres NM, Lin AA, Jarve M, King RJ, Kutuev I, et al Distinguishing the co-ancestries of haplogroup G Y-chromosomes in the populations of Europe and the Caucasus. European journal of human genetics: EJHG. 2012;20(12):1275–82. 10.1038/ejhg.2012.86 22588667 PMC3499744

[45] Saillard J, Forster P, Lynnerup N, Bandelt H-J, Nørby N. mtDNA Variation among Greenland Eskimos: The Edge of the Beringian Expansion. Am J Hum Genet. 2000; 67(3):718–26. 10924403 10.1086/303038 PMC1287530

[46] Sanchez-Diz P, Alves C, Carvalho E, Carvalho M, Espinheira R, Garcia O, et al Population and segregation data on 17 Y-STRs: results of a GEP-ISFG collaborative study. International journal of legal medicine. 2008;122(6):529–33. 10.1007/s00414-008-0265-z 18651159

[47] Sengupta S, Zhivotovsky LA, King R, Mehdi SQ, Edmonds CA, Chow C-ET, et al Polarity and Temporality of High-Resolution Y-Chromosome Distributions in India Identify Both Indigenous and Exogenous Expansions and Reveal Minor Genetic Influence of Central Asian Pastoralists. Am J Hum Genet. 2006;78:203–21. 10.1086/499411 PMC1380230 16400607

[48] Torlakian BG. Ethnography of Hamshen Armenians Ethnography and Folklore. 13 Yerevan 1981 p. 24–111 (In Armenian).

[49] Wei W, Ayub Q, Chen Y, McCarthy S, Hou Y, Carbone I, et al A calibrated human Y-chromosomal phylogeny based on resequencing. Genome Res. 2013;23(2):388–95. 10.1101/gr.143198.112 23038768 PMC3561879

[50] Xue Y, Wang Q, Long Q, Ng BL, Swerdlow H, Burton J, et al Human Y chromosome base-substitution mutation rate measured by direct sequencing in a deep-rooting pedigree. Curr Biol. 2009;19(17):1453–7. 10.1016/j.cub.2009.07.032 19716302 PMC2748900

[51] Xue Y, Zerjal T, Bao W, Zhu S, Shu Q, Xu J, et al Male demography in East Asia: a north-south contrast in human population expansion times. Genetics. 2006;172(4):2431–9. 16489223 10.1534/genetics.105.054270 PMC1456369

[52] Yunusbayev B, Metspalu M, Jarve M, Kutuev I, Rootsi S, Metspalu E, et al The Caucasus as an asymmetric semipermeable barrier to ancient human migrations. Molecular biology and evolution. 2012;29(1):359–65. 10.1093/molbev/msr221 21917723

[53] Zerjal T, Xue Y, Bertorelle G, Wells RS, Bao W, Zhu S, et al The Genetic Legacy of the Mongols. Am J Hum Genet. 2003;72:717–21. 12592608 10.1086/367774 PMC1180246

[54] Zhivotovsky LA, Underhill PA, Cinniog˘lu C, Kayser M, Morar B, Kivisild T, et al The Effective Mutation Rate at Y Chromosome Short Tandem Repeats, with Application to Human Population-Divergence Time. Am J Hum Genet. 2004;74:50–61. 14691732 10.1086/380911 PMC1181912

[55] Zhong H, Shi H, Qi XB, Xiao CJ, Jin L, Ma RZ, et al Global distribution of Y-chromosome haplogroup C reveals the prehistoric migration routes of African exodus and early settlement in East Asia. J Hum Genet. 2010;55(7):428–35. 10.1038/jhg.2010.40 20448651

