# Supplementary figures and images for: Deep Phylogenetic Analysis of Haplogroup G1 Provides Estimates of SNP and STR Mutation Rates on the Human Y-Chromosome and Reveals Migrations of Iranic Speakers
Source: PLoS One. 2015 Apr 7;10(4):e0122968. doi: 10.1371/journal.pone.0122968 (PMC4388827; doi:10.1371/journal.pone.0122968)

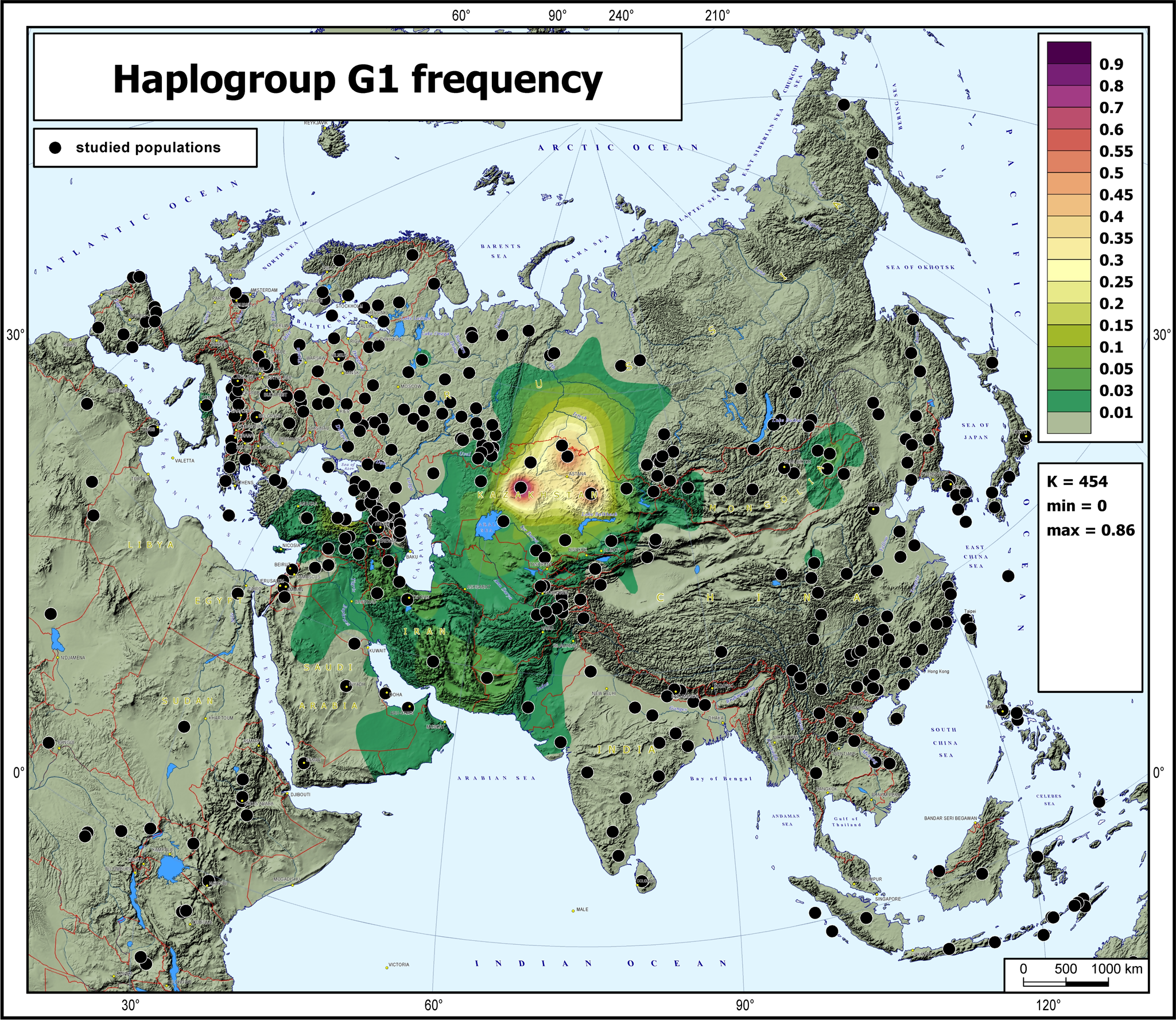

Supplement: S1 Fig — This scale is typically used in the GeneGeo software for frequency distribution maps of all haplogroups, thus allowing easy comparisons of different maps. The black points represent the populations analyzed. Abbreviations in the statistical legend indicate the following: K, number of the populations studied; MIN and MAX, the minimal and maximum frequencies on the map. (TIFF) [file pone.0122968.s002.tiff]

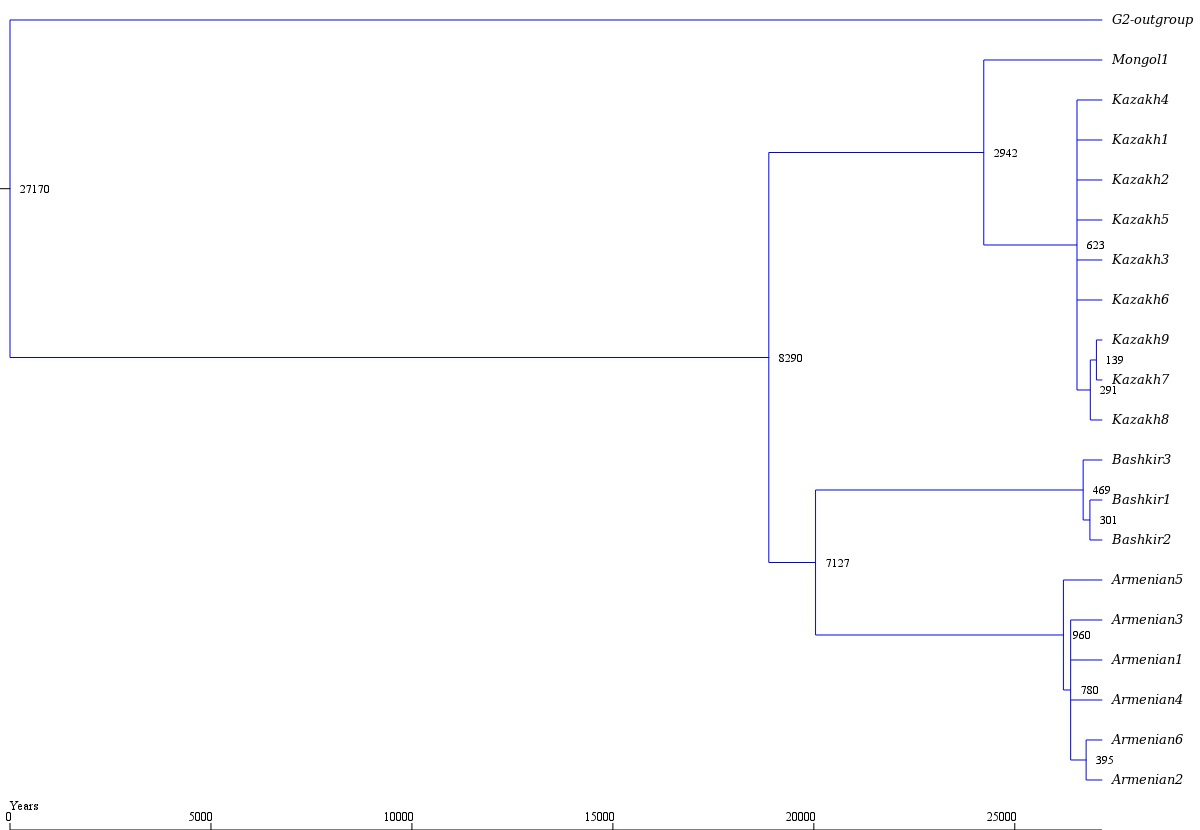

Supplement: S3 Fig — The tree is based on the high quality filtered dataset from this study consisting of 20 samples and 636 SNPs. The tree was created in the BEAST software. The mean age estimates are shown for all branches. (TIFF) [file pone.0122968.s004.tiff]

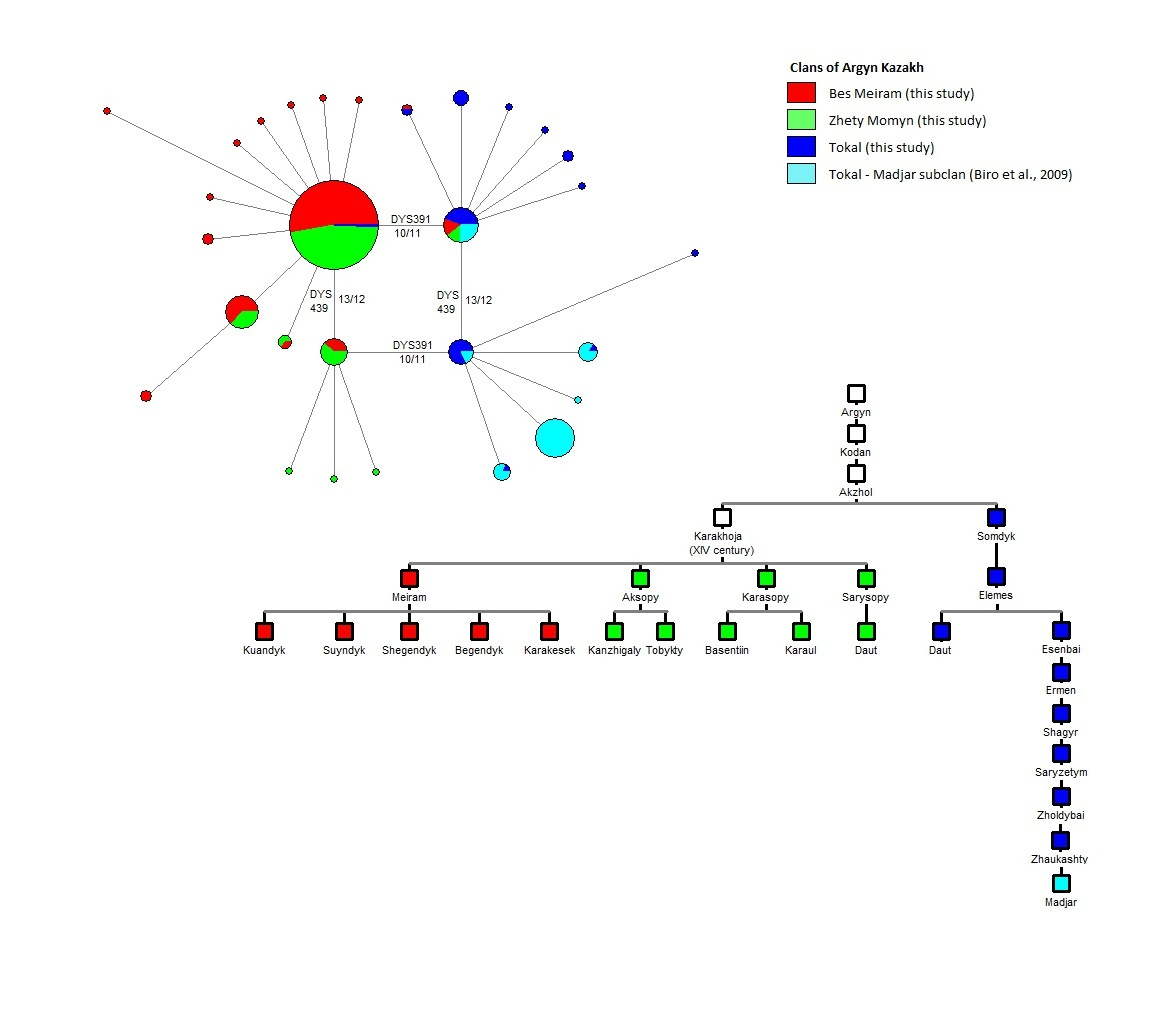

Supplement: S4 Fig — Data on haplogroup G1 Y-STRs in the Argyn tribe of the Kazakh clan came from both this study and [5]. 10-STRs haplotypes were used. The genealogy of the early generations of the Argyn tribe shows partitioning into clans. Members of each clan are color-coded in both network and genealogy. (TIFF) [file pone.0122968.s005.tiff]
