# Supplementary material for: Deep Phylogenetic Analysis of Haplogroup G1 Provides Estimates of SNP and STR Mutation Rates on the Human Y-Chromosome and Reveals Migrations of Iranic Speakers
Source: PLoS One. 2015 Apr 7;10(4):e0122968. doi: 10.1371/journal.pone.0122968 (PMC4388827; doi:10.1371/journal.pone.0122968)
Supplement: S2 Fig — The tree is based on the high quality filtered dataset from this study consisting of 20 samples and 636 SNPs. The Build 37 coordinates of the SNPs are shown along branches. ISOGG marker names are shown in red. Further details of these mutations are reported in S4 Table. (PDF) [file pone.0122968.s003.pdf]

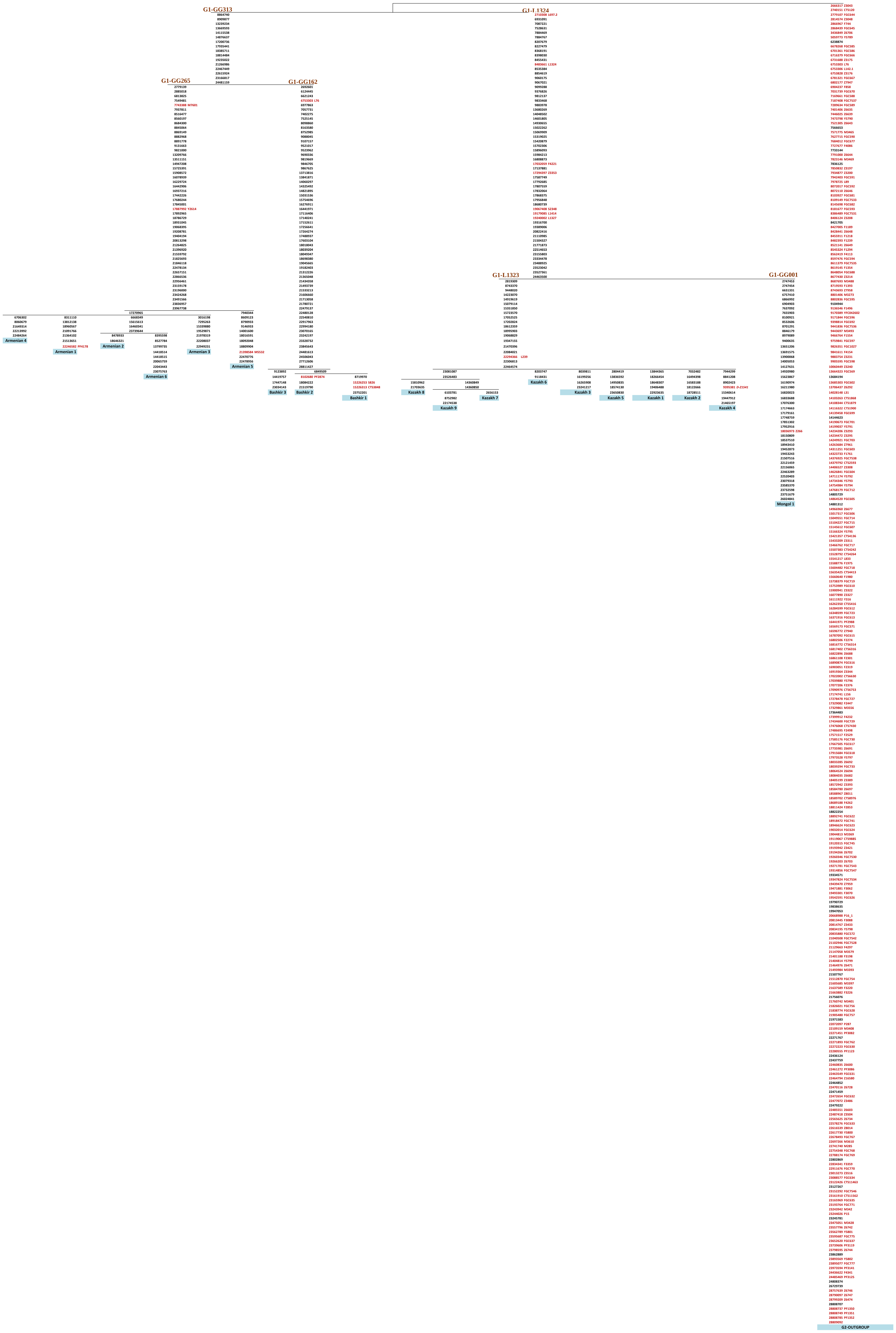
G1-GG313
G1-L1324
G1-GG265
G1-GG162
G1-L1323
G1-GG001
Armenian 4
Armenian 1
Armenian 2
Armenian 3
Armenian 6
Armenian 5
Bashkir 3
Bashkir 2
Bashkir 1
Kazakh 8
Kazakh 9
Kazakh 7
Kazakh 6
Kazakh 3
Kazakh 5
Kazakh 1
Kazakh 2
Kazakh 4
Mongol 1
G2-OUTGROUP
